# Evaluation of MRI‐only based online adaptive radiotherapy of abdominal region on MR‐linac

**DOI:** 10.1002/acm2.13838

**Published:** 2022-11-08

**Authors:** Katri Nousiainen, Grichar Valdes Santurio, Nils Lundahl, Rickard Cronholm, Carl Siversson, Jens M. Edmund

**Affiliations:** ^1^ Department of Physics University of Helsinki Helsinki Finland; ^2^ HUS Cancer Center Helsinki University Hospital and University of Helsinki Helsinki Finland; ^3^ HUS Medical Imaging Center Helsinki University Hospital and University of Helsinki Helsinki Finland; ^4^ Department of Oncology, Radiotherapy Research Unit, Herlev and Gentofte Hospital Copenhagen University Herlev Denmark; ^5^ Spectronic Medical AB Helsingborg Sweden; ^6^ Nils Bohr Institute Copenhagen University Copenhagen Denmark

**Keywords:** MR‐linac, MRI‐only, online adaptive radiotherapy, synthetic CT

## Abstract

**Purpose:**

A hybrid magnetic resonance linear accelerator (MRL) can perform magnetic resonance imaging (MRI) with high soft‐tissue contrast to be used for online adaptive radiotherapy (oART). To obtain electron densities needed for the oART dose calculation, a computed tomography (CT) is often deformably registered to MRI. Our aim was to evaluate an MRI‐only based synthetic CT (sCT) generation as an alternative to the deformed CT (dCT)‐based oART in the abdominal region.

**Methods:**

The study data consisted of 57 patients who were treated on a 0.35 T MRL system mainly for abdominal tumors. Simulation MRI‐CT pairs of 43 patients were used for training and validation of a prototype convolutional neural network sCT‐generation algorithm, based on HighRes3DNet, for the abdominal region. For remaining test patients, sCT images were produced from simulation MRIs and daily MRIs. The dCT‐based plans were re‐calculated on sCT with identical calculation parameters. The sCT and dCT were compared in terms of geometric agreement and calculated dose.

**Results:**

The mean and one standard deviation of the geometric agreement metrics over dCT–sCT‐pairs were: mean error of 8 ± 10 HU, mean absolute error of 49 ± 10 HU, and Dice similarity coefficient of 55 ± 12%, 60 ± 5%, and 82 ± 15% for bone, fat, and lung tissues, respectively. The dose differences between the sCT and dCT‐based dose for planning target volumes were 0.5 ± 0.9%, 0.6 ± 0.8%, and 0.5 ± 0.8% at *D*_2%_, *D*_50%_, and *D*_98%_ in physical dose and 0.8 ± 1.4%, 0.8 ± 1.2%, and 0.6 ± 1.1% in biologically effective dose (BED). For organs‐at‐risk, the dose differences of all evaluated dose–volume histogram points were within [–4.5%, 7.8%] and [–1.1 Gy, 3.5 Gy] in both physical dose and BED.

**Conclusions:**

The geometric agreement metrics were within typically reported values and most average relative dose differences were within 1%. Thus, an MRI‐only sCT‐based approach is a promising alternative to the current clinical practice of the abdominal oART on MRL.

## 1 INTRODUCTION

A hybrid magnetic resonance linear accelerator (MRL) system can be used for acquiring magnetic resonance (MR) images that serve as a basis for image‐guided radiation therapy (IGRT).^1^ The MR imaging (MRI) provides many benefits for radiation therapy (RT): the high soft‐tissue contrast enables improved delineation of target volumes and organs‐at‐risk (OARs) and precise position verification of non‐bony tissues in IGRT as compared to a computed tomography (CT)‐based alternative.^2^ In addition, the MRL‐acquired images can be used for online adaptive radiation therapy (oART), where the RT structures are re‐contoured and the RT plan is re‐optimized, while the patient is on the treatment couch.^3^ The MRL system also offers a possibility of tracking the target volume during irradiation with automatic beam hold if the target volume moves outside set boundaries.^4^, ^5^ Because day‐to‐day organ filling differences and physiological movements in the abdominal and pelvic regions can be substantial, the MRL systems are often utilized for treatments in these regions.^6^, ^7^ Furthermore, the contrast of the conventional CT‐based technique is limited in the abdominal and pelvic regions that are largely composed of soft tissues.^3^ Altogether, the benefits of MRL allow reduced margins around the target volume, which can decrease toxicity in the adjacent OARs.^7^, ^8^

A limitation of MRI is that, unlike CT scans, the MR images do not contain information about the electron density (ED) of the tissues, which is required for optimizing and calculating dose distributions. Currently, a common clinical practice of MRI‐guided oART is to obtain a planning CT (pCT) in advance for the dose calculation.^9^, ^10^ On a commercial MRL system, the pCT can be non‐rigidly registered to MRL‐acquired images resulting in a deformed CT (dCT), which then serves as an ED map for the dose calculation possibly in combination with manually assigned bulk ED.^10^ However, the organ fillings and the air pocket positions can differ between the pCT and MRI acquisitions, yet the deformable image registration cannot always compensate for this mismatch.^11^ The image‐registration issues may require manual corrections, which can be time‐consuming. If corrections are necessary during the treatment adaption, the consequent delay can cause discomfort for the patient and reduce the accuracy between the imaged and the actual patient anatomy, which again can compromise the benefit of the adaptive workflow.^7^, ^12^ Moreover, even if the CT‐MRI co‐registration is successful, the registration is accompanied by an uncertainty of 2–3 mm.^8^

One way to overcome the CT‐to‐MRI challenges in the MRI‐based oART is to replace the dCT with a synthetic CT (sCT) generated directly from the MRL‐acquired images. Previously, these so‐called MRI‐only methods have been proposed for conventional RT, especially in the pelvis and brain.^13^, ^14^ Lately, the MRI‐only sCT‐generation methods using deep learning (DL), especially convolutional neural networks (CNN), have gained popularity.^15^ For example, U‐Net architectures and modified generative adversarial network frameworks have shown promising results.^2^, ^8^, ^15^ The CNN‐based sCT‐generation from 1.5 or 3 T MR images have been successfully implemented for the RT planning (RTP) of the prostate^16^ and head‐and‐neck,^17^ as well as the proton therapy of the prostate,^18^ the brain,^19^ and pediatric abdomen.^20^ In addition, a DL‐based sCT approach for a single fraction breast RT on a 1.5 T MRL has been studied and concluded as an applicable alternative.^21^ The DL‐based approaches have also been used to generate sCT images from low‐field (0.35 T) MRL‐acquired images in the pelvic region,^22^ the abdominal region,^23^, ^24^ and both.^25^ These studies reported average global mean absolute errors (MAE) of 29.7–54.3 HU between the reference CT and sCT in the pelvic region and 35.6–94.1 HU in the abdominal region and target coverage and dose‐to‐OARs differences less than 1% between the proposed and conventional methods with liver and pancreas patients.^22^, ^23^, ^24^, ^25^

Regardless of the popularity of the CNN‐based sCT‐generation approaches, the sCT‐generation from low‐field MRL‐acquired images, together with the use of sCT for abdominal region and for oART have only been sparsely reported. Here, we evaluate the geometric and dosimetric agreement of an sCT‐based approach in comparison to the existing dCT‐based workflow for abdominal oART using a 0.35 T MRL system. The aim is to evaluate an MRI‐only approach as an alternative to the current clinical practice.

## 2 MATERIALS AND METHODS

### 2.1 Patient material

In total 57 patients were included in this retrospective study. All patients had received RT for thoracic, abdominal, or pelvic cancer (e.g., lung, pancreas, liver, adrenal gland, prostate, or lymph node target) between June 2019 and August 2021 on a 0.35 T MRL (MRIdian Linac, ViewRay Technologies Inc., Oakwood Village, OH, USA) at Herlev and Gentofte Hospital, Herlev, Denmark. We included 43 patients in the training and validation groups of a prototype sCT‐generation algorithm and preserved the remaining 14 patients for clinical evaluation. A single thoracic patient was later excluded from the clinical testing group due to a lack of tumor presentation on the lungs in the generated sCT, leaving 13 test patients of whom 11 had received oART. Further details of the patient groups are given in Table 1.

**TABLE 1:** The patient material

	All	Training and validation	Clinical testing
Number of patients	57	43	13 + 1^*^
Average age (1SD) (years)	70 (11)	69 (11)	72 (9)
Age (range) (years)	42–86	42–86	48–82
Number of males/females	41/15	43/11	7/4 + 1^*^
Number of abdominal cases	42	32	9
Number of pelvic cases	9	6	3
Number of thoracic cases	6	5	1^*^

*Note*: One thoracic case was excluded from the clinical testing data (marked with asterisk “*”). 1SD = one standard deviation.

### 2.2 Imaging material

We used both the simulation MRI (SIM‐MRI) and the corresponding clinically used pCT of each included patient along with the daily MRIs of the 13 test patients. The pCT scans were acquired prior to the SIM‐MRI with three different CT scanners using 120 kV tube voltage and 2 mm slice thickness, and in a similar position as in which RT was delivered.

The SIM‐MRIs and the daily MRIs were acquired on the 0.35 T MRL system. The MRIs were obtained using a True Fast Imaging with Steady State Precession (TRUFI) sequence, which is a balanced steady‐state free precession sequence yielding a T2/T1‐weighted contrast.^10^ The MR images had a 1.5 mm × 1.5 mm in‐plane resolution, 1.5–3.0 mm slice thicknesses, and 80–288 slices.

In the test data, two of the 13 patients received two independent RT courses, resulting in 15 different RT courses, and consequently, 15 SIM‐MRIs obtained prior to the first fraction. The 15 RT courses each consisted of 3–20 fractions with most courses containing five fractions (see Table 2). From all the RT courses of the test patients, 96 daily MRIs were acquired resulting in 111 individual MRIs together with SIM‐MRIs.

**TABLE 2:** The prescribed doses of the test patients

Target area	Number of fractions	Fractional dose (Gy)	Total dose (Gy)
Adrenal gland (*N* = 2)	5	10	50
Pancreas (*N* = 4)	3	8	24
Liver (*N* = 4)	3	15	45
	5	15	75
	5	10	50
	6	8	48
Stomach (*N* = 1)	5	5	25
Lymph nodes (*N* = 2)	5	10	50
Prostate (*N* = 2)	20	3	60

*Note*: The different anatomical targets and their number (*N*) within the test data with the total dose, the fractional dose, and the number of fractions for each case.

The dCTs were generated from the pCT scans by performing a deformable registration between the pCT and a corresponding SIM‐MRI or daily MRI in ViewRay treatment planning system (TPS) (version 5.4.0.97). After the image registration, the dCT had the same image dimensions as the target MRI.

### 2.3 Synthetic CT‐generation

The original pCT and SIM‐MRI of 43 patients were used for the training and validation of a CNN‐based prototype sCT‐generation algorithm for the abdominal region. Prior to algorithm training, the pCT and SIM‐MR were deformably registered using *elastix*‐toolbox.^26^, ^27^ The algorithm utilized a high‐resolution 3D CNN architecture modified from HighRes3DNet, which uses dilated convolutions to incorporate volumetric information from neighborhoods of varying sizes.^28^ The HighRes3DNet has some benefits in terms of spatial perception and retaining spatial information while avoiding a vanishing gradients problem efficiently. The HighRes3DNet was modified to produce MRI‐to‐sCT transfer function coefficients as the output.^29^

For the clinical testing of the prototype algorithm, an sCT was generated from each of the 111 TRUFI MRIs (15 SIM‐MRIs and 96 daily MRIs). The hardware used for the sCT production consisted of Intel Core i7 (Intel Corporation, Santa Clara, CA, USA) central processing unit with 64 Gb of RAM and an NVIDIA TITAN RTX (NVIDIA Corporation, Santa Clara, CA, USA) graphics processing unit (GPU) with 24 Gb of memory.

**FIGURE 1:**
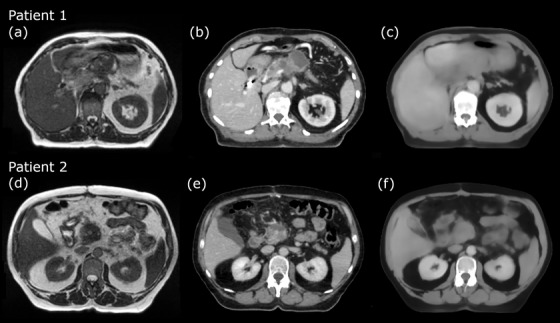
Examples of simulation magnetic resonance imaging (MRI), deformed computed tomography (CT), and synthetic CT. Corresponding axial slices at the isocenter level of two patients with (a and d) the simulation MRI, (b and e) the deformed CT, and (c and f) the synthetic CT produced with the prototype abdominal. The overall geometric agreement was among the worst for patient 1 and among the best for patient 2

Examples of the SIM‐MRI, dCT, and sCT produced with the prototype abdominal algorithm are presented in Figure 1 for two patients. These two patients had one of the worst (patient 1) and one of the best (patient 2) overall geometric agreement of the dCT and sCT, and they had both received RT for pancreatic tumors.

### 2.4 Geometric evaluation

We evaluated the geometric agreement between corresponding sCT and dCT pairs using several similarity metrics in MATLAB v2020b (The MathWorks Inc., Natick, MA, USA). As pre‐processing, we excluded the background noise through masking. The delineated body structure from the RT plan did not always follow the body outline, especially at the edges of the field‐of‐view (FOV), thus we set a threshold for both sCT and dCT >–950 HU to obtain two additional body masks. We used the intersection of the two body masks and the delineated body structure as the volume‐of‐interest (VOI) in the evaluation. In addition, the three most cranial and caudal slices were left out from the evaluation, due to blurring in sCT images caused mainly by MRI edge effects such as limited FOV. We also allocated HU values of water to the air pockets in the dCT if there was no corresponding air pocket in the sCT: areas where the HU values were less than –500 HU in the dCT and >–200 HU in the sCT were set to 0 HU as previously reported.^30^ This process was performed to compensate for differences not caused by the sCT‐generation algorithm, as the air pockets usually differ between a daily MRI and a corresponding dCT.

We determined the voxel‐wise mean error (ME):

(1) $$\mathrm{ME}\;=\frac{1}{N}\;\sum_{i\;=\;1}^{N}\left(\mathrm{dC}T_{i}-\mathrm{sC}T_{i}\right)$$

where *N* is the number of voxels inside the VOI and dCT*_i_* and sCT*_i_* are the *i*th voxel in the dCT and the sCT, respectively.

We also calculated the voxel‐wise MAE for the whole‐body VOI:

(2) $$\mathrm{MAE}\;=\frac{1}{N}\;\sum_{i\;=\;1}^{N}\left|\mathrm{dC}T_{i}-\mathrm{sC}T_{i}\right|$$

where *N* is the number of voxels inside VOI and dCT*_i_* and sCT*_i_* are the *i*th voxel. In addition, MAE was calculated separately in 20 HU intervals from –1000 to 1500 HU.^31^

We calculated the Dice similarity coefficient (DSC) for bone (HU value >150 HU), fat (HU value >–100 and <–20), and lungs (HU value <–700 HU) within the body outline:

(3) $$\mathrm{DSC}\;=\;100\%\;\times \frac{2\left(V_{\mathrm{dCT}}\cap V_{\mathrm{sCT}}\right)}{V_{\mathrm{dCT}}+V_{\mathrm{sCT}}}$$

where *V*_dCT_ and *V*_sCT_ are the volumes of the tissue masks in the dCT and the sCT, respectively, and $V_{\mathrm{dCT}}\cap V_{\mathrm{sCT}}$ is the intersection of those volumes. A DSC of 100% implies complete overlap of volumes, and the DSC of 0% implies no overlap. The thresholds for the tissue masks were chosen for the fat and the lungs according to the corresponding relative ED (RED) in the ICRU report 46,^32^ as 0.93–0.98 and 0.26, respectively. For the bone, RED of 1.2 was obtained from the ViewRay TPS. REDs were converted to the HU values using the generic calibration curve available in the ViewRay TPS. DSC_lung_ was calculated only for the abdominal cases due to little to none lung tissue in the pelvic MRIs.

### 2.5 Dosimetric evaluation

#### 2.5.1 Treatment planning and delivery

The RTP of both the base plans (i.e., the RT plan generated based on a SIM‐MRI prior to the first fraction) and the daily adaptive plans was performed using the ViewRay TPS. All plans were optimized and delivered as step‐and‐shoot intensity‐modulated RT with 6 MV flattening filter‐free photon beams. In the test data, 13 RT courses were stereotactic body RT (SBRT) and two were prostate cases. The 15 RT courses included 17 different planning target volumes (PTVs). The prescribed doses varied across the test patients and are given in Table 2.

Of the 15 RT courses, 11 were oART and four were conventional RT, where the daily MRI was used for positioning and the base plan was delivered without modifications. The 11 oART courses resulted in 62 daily adaptive plans that were re‐contoured and re‐optimized online. Of the daily adaptive plans, 46 had been calculated using a dCT as the ED map during the RT course, but 16 daily adaptive plans had been calculated using a rigidly registered CT; we re‐calculated those 16 plans offline using the dCT instead. We also generated daily dose distributions for the 34 fractions of the four conventional RT courses by re‐calculating the base plan on a dCT registered to the daily MRI; however, neither re‐contouring nor re‐optimization was performed for these cases.

For the dosimetric evaluation, we re‐calculated the dCT‐based plans using the sCT as the ED map within the ViewRay TPS while keeping all plan parameters, especially monitor units, unchanged in comparison to the dCT‐based calculation. In the Monte Carlo (MC) calculations, the standard uncertainty was 0.5% with the grid resolution of 0.20 cm. The ViewRay TPS allows MC calculation with or without the presence of the magnetic field; all the calculations were performed taking the magnetic field into account.

#### 2.5.2 Dose accumulation and reporting

We performed dose accumulation using both the dCT‐based and sCT‐based daily adaptive dose distributions. The dose accumulation was accomplished through deformable registration using 3D Slicer (version 4.11)^33^ and the *elastix*‐toolbox within 3D Slicer. All the daily MRIs were first manually roughly aligned with a corresponding SIM‐MRI, then rigidly and deformably registered to the SIM‐MRI using *elastix*. Parameter file par0059^34^ was used for the rigid and deformable registrations with a mask covering the clinically interesting volume (i.e., high‐dose area and delineated structures). The same transforms were then applied to the dCT‐based and sCT‐based daily dose distributions and the daily RT structures. The SIM‐MRI served as the common frame‐of‐reference, that is, all the daily MR images and dose distributions were transformed back to this frame. We verified the registration results through visual inspections and by calculating a DSC score of the planned versus the daily gross tumor volume (GTV) or clinical target volume (CTV) if no GTV was available. The mean DSC over GTVs and CTVs was 80% after the registrations.

We compared the dose distributions in MATLAB. Using clinically relevant structures delineated on the SIM‐MRI as binary masks, fractional doses were extracted from both the dCT and sCT dose distributions for each structure in turn. The voxel‐wise sum of the fractional doses resulted in the accumulated dose. We calculated the dose–volume histograms (DVH) from the voxel‐wise doses and compared the DVH values at clinically relevant points. For PTVs, the DVH points describing the target coverage were the dose to 2%, 50%, and 98% of the volume (i.e., *D*_2%_, *D*_50%_, and *D*_98%_) according to ICRU report 83.^35^ In addition, we considered some other target coverage metrics and different dose constraints according to the local clinical practice, which are listed in Tables 4 and 5 for the target volumes and the OARs, respectively. We calculated the dose difference at each considered DVH point relative to the dCT‐based dose.

The same procedure was repeated but including the effect of tissue fraction sensitivity by converting the fractional physical doses to a biologically effective dose (BED) before dose accumulation according to:

(4) $$\mathrm{BED}\;=\;D\left(1+\frac{d}{\alpha /\beta }\right)$$

where *D* is the total dose, *d* is the dose per fraction received by a voxel, and *α*/*β* is a tissue‐specific value obtained from Joiner and van der Kogel,^36^ or generically set to 10 for the target volumes and 3 for OARs if not otherwise specified. This was done to investigate if large dose differences obtained in one fraction could be compensated for in another fraction before accumulating the BED. The DVH comparison was also performed between individual daily dCT and sCT pairs.

Finally, we performed gamma analysis^37^ for the accumulated sCT‐based (evaluation) and dCT‐based (reference) physical doses using the 3D Slicer RT‐plugin, SlicerRT.^38^ Local 3D gamma indices were calculated with 1%/1 mm, 2%/2 mm, and 3%/3 mm distance‐to‐agreement/dose‐difference tolerance criteria applying an exclusion threshold at 10% of the maximum dose.

## 3 Results

Figure 1 shows corresponding axial slices of the SIM‐MRI, dCT, and sCT for two patients. The sCTs were smoother compared to the dCTs, which is characteristic for sCT‐generation. The outermost cranial–caudal slices of the sCT were increasingly blurred, due to zero‐padding in CNN (i.e., adding voxels containing zeroes around the original volume) and the nature of data in the MR images, where the FOV did not always cover the whole outermost axial slices. Smaller bony structures such as ribs are not visible in the sCT, in contrast to larger bony structures such as vertebrae that are clearly represented. The abdominal algorithm was not able to reproduce a lung tumor in the sCT for a single test case. For 95 calculations, the generation of an sCT took on average 158.2 s with one standard deviation (1SD) of 30.3 s and a range from 129 to 224 s. Each produced sCT had the same image dimensions as the input MRI, and hence, the same as the corresponding dCT.

### 1 Geometric agreement

Table 3 shows the results for the geometric and HU‐agreement of the dCT and sCT pairs as the average and 1SD of the performance metrics for different anatomies included in the investigation. The ME values were close to 0 HU and the MAE values were around 50 HU. The DSC scores were mostly above 50% with DSC_lung_ for the abdominal cases being the highest (>80%) and DSC_bone_ for the abdominal cases being the lowest (<50%).

**TABLE 3:** Values for the geometric and HU‐agreement metrics between deformed computed tomography (dCT) and synthetic CT (sCT)

Cases	ME (HU)	MAE (HU)	DSC_bone_ (%)	DSC_fat_ (%)	DSC_lung_ (%)
Abdominal (*N* = 63)	8 (13)	56 (7)	47 (7)	58 (4)	82 (15)
Pelvic (*N* = 48)	–4 (1)	45 (5)	64 (4)	57 (3)	–
All (*N* = 111)	8 (10)	49 (10)	55 (12)	60 (5)	–

*Note*: Values are given as averages (one standard deviation). DSC_lung_ was calculated only for the abdominal cases.

Abbreviations: DSC, Dice similarity coefficient; MAE, mean absolute error; ME, mean error; *N*, the number of the dCT–sCT‐pairs per set.

**FIGURE 2:**
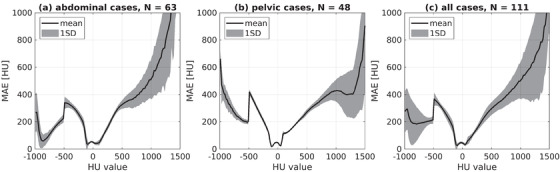
The mean absolute error (MAE) between deformed computed tomography (dCT) and synthetic CT (sCT) in 20 HU intervals. The MAE is averaged over (a) the abdominal cases, (b) the pelvic cases, and (c) all the cases. The gray area defines one standard deviation (1SD). The drop at –500 HU shows the threshold where air pockets were set to 0 HU

Figure 2 shows MAE determined in 20 HU intervals as a function of the HU value (midpoint of the interval) and averaged over the abdominal, pelvic, and all cases. MAE was less than 100 HU around 0 HU, but increased especially toward higher positive HU values.

### 2 Dosimetric agreement

**FIGURE 3:**
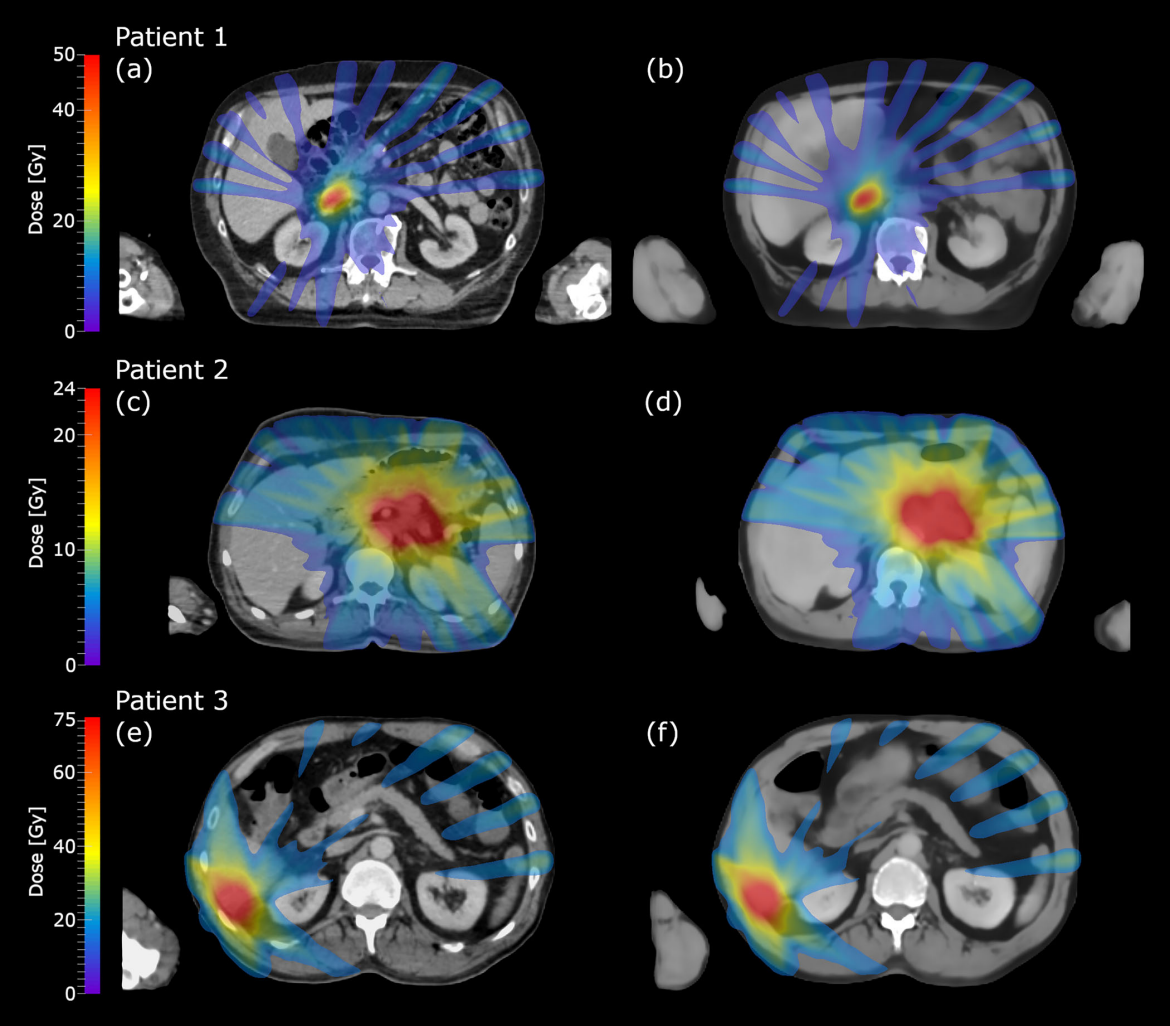
Accumulated dose distributions on deformed computed tomography (dCT) and synthetic CT (sCT). The accumulated physical dose distributions calculated for three patients on dCT (a, c, and e) and sCT (b, d, and f). The prescribed doses are 50 Gy in five fractions to adrenal gland for patient 1, 24 Gy in three fractions to pancreas for patient 2, and 75 Gy in five fractions to liver for patient 3. The dose distributions are thresholded at 5, 3, and 15 Gy for the patients 1–3, respectively. The overall dose differences were among the best for patients 1 and 2, and among the worst for patient 3.

Figure 3 shows examples of accumulated dose distributions in physical dose for three patients calculated both on dCT and sCT. Patients 1 and 2 had overall one of the smallest overall dose differences, whereas patient 3 had overall one of the largest dose differences. The all three example patients had received RT with different prescribed doses for tumors at different anatomical locations.

Table 4 shows the relative dose difference between the dCT and sCT for PTV, GTV, and CTV at different DVH points for accumulated physical dose and BED. All the dose differences at *D*_2%_, *D*_50%_, and *D*_98%_ for PTVs were within –0.5% and 2.6% in physical dose. The mean differences were less than ±0.6% and ±0.9% in physical dose and BED, respectively, for all the evaluated DVH points of the target coverage. The largest differences were observed at *D*_99%_ for PTVs (and *D*_0.1cc_ for GTVs) of the SBRT cases. The absolute dose differences were between –0.5 and 1.6 Gy in physical dose and between –1.6 and 6.1 Gy in BED for PTVs. The dose differences were larger in BED than in physical doses. The dose differences between the daily dCT and sCT pairs are given for target volumes as shown in Table S1; the mean differences were less than ±0.5% and ±0.8% in physical dose and BED, respectively, and all values were within –3.1% and 4.9% in physical dose.

**TABLE 4:** Dose differences between deformed computed tomography (dCT) and synthetic CT (sCT) for target volumes at dose–volume histogram (DVH) points

Cases	Target	DVH point	Physical dose difference (%)		BED difference (%)	
			Mean (1SD)	[min, max]	Mean (1SD)	[min, max]
All	PTV (*N* = 17)	*D*_2%_	0.5 (0.9)	[–0.5, 2.6]	0.8 (1.4)	[–0.8, 3.9]
		*D*_50%_	0.6 (0.8)	[–0.5, 2.6]	0.8 (1.2)	[–0.8, 4.0]
		*D*_98%_	0.5 (0.8)	[–0.5, 2.6]	0.6 (1.1)	[–1.3, 3.7]
SBRT	PTV (*N* = 15)	*D*_99%_	0.4 (1.0)	[–0.8, 3.2]	0.6 (1.4)	[–1.2, 4.0]
	GTV (*N* = 15)	*D*_0.1cc_	0.4 (1.1)	[–1.7, 2.6]	0.6 (1.8)	[–2.5, 4.2]
		*D*_mean_	0.6 (0.9)	[–0.7, 2.7]	0.9 (1.4)	[–1.0, 4.1]
		*D*_99%_	0.5 (0.9)	[–0.9, 2.3]	0.7 (1.4)	[–1.3, 3.3]
Prostate	PTV (*N* = 2)	*D*_2%_	0.1 (0.2)	[0.0, 0.3]	0.1 (0.3)	[–0.1, 0.3]
		*D*_50%_	0.2 (0.1)	[0.1, 0.2]	0.2 (0.1)	[0.2, 0.3]
		*D*_98%_	0.2 (0.2)	[0.1, 0.4]	0.3 (0.2)	[0.2, 0.4]
	CTV (*N* = 2)	*D*_98%_	0.2 (0.0)	[0.2, 0.2]	0.2 (0.1)	[0.2, 0.3]

*Note*: The mean with one standard deviation (1SD) and range for both physical dose and biologically effective dose (BED) difference are presented. The values are given in percent relative to the dCT‐based dose.

Abbreviations: CTV, clinical target volume; GTV, gross tumor volume; PTV, planning target volume; SBRT, stereotactic body radiation therapy.

Table 5 shows the relative dose difference between the dCT and sCT for OARs at different DVH points with accumulated physical dose and BED. All the relative dose differences of OARs at clinically relevant DVH points were between –4.4% and 5.7% in physical dose (mean ± 1SD: 0.3 ± 1.2%) and between –4.5% and 7.8% in BED (mean ± 1SD: 0.5 ± 1.7%). The largest relative dose differences were observed at *D*_0.03cc_ for spinal cord, *D*_0.5cc_ for stomach, and *D*_0.5cc_ and *D*_5cc_ for duodenum. However, all the absolute dose differences were between –0.2 and 0.9 Gy in physical dose and between –1.1 and 3.5 Gy in BED for OARs. Overall, the doses were mostly higher in the dCT calculations as compared to the sCT calculations, and in BED rather than in physical doses. The dose differences between the daily dCT and sCT pair are given for OARs as shown in Table S2; the dose differences were between –6.6% and 8.7% in physical dose (mean ± 1SD: 0.3 ± 1.4%) and between –6.9% and 12.0% in BED (mean ± 1SD: 0.4 ± 1.9%).

**TABLE 5:** Dose differences between deformed computed tomography (dCT) and synthetic CT (sCT) for organs‐at‐risk at dose–volume histogram (DVH) points

Structure	DVH point	Physical dose difference (%)		BED difference (%)	
		Mean (1SD)	[min, max]	Mean (1SD)	[min, max]
Spinal cord (*N* = 11)	*D*_0.03cc_	1.2 (1.7)	[–0.7, 4.8]	1.5 (2.4)	[–1.3, 6.1]
Kidney L (*N* = 10)	*D*_10%_	0.3 (0.7)	[–0.7, 1.8]	0.4 (0.9)	[–0.9, 2.4]
	*D*_25%_	0.2 (1.4)	[–3.1, 2.0]	0.3 (1.6)	[–3.2, 2.7]
	*D*_mean_	0.2 (0.9)	[–1.3, 1.9]	0.3 (1.1)	[–1.2, 2.5]
Kidney R (*N* = 9)	*D*_10%_	0.5 (1.3)	[–0.8, 3.5]	0.6 (1.5)	[–1.1, 3.8]
	*D*_25%_	0.0 (1.5)	[–2.4, 2.7]	0.0 (1.8)	[–2.6, 3.1]
	*D*_mean_	0.1 (1.4)	[–1.8, 3.0]	0.2 (1.7)	[–1.6, 3.8]
Pancreas (*N* = 3)	*D*_mean_	0.9 (1.1)	[0.2, 2.2]	1.5 (2.1)	[0.1, 3.9]
Liver (*N* = 9)	*D*_mean_	0.3 (0.4)	[–0.3, 0.9]	0.7 (0.9)	[–0.4, 2.4]
Heart (*N* = 2)	*D*_0.03cc_	0.3 (2.0)	[–1.1, 1.7]	0.7 (2.9)	[–1.4, 2.8]
	*D*_15cc_	–0.3 (0.9)	[–0.9, 0.4]	–0.2 (0.8)	[–0.7, 0.4]
Great vessels (*N* = 15)	*D*_0.5cc_	0.5 (1.1)	[–0.6, 3.0]	0.9 (1.6)	[–0.7, 4.7]
Esophagus (*N* = 9)	*D*_0.5cc_	–0.5 (1.7)	[–3.9, 2.6]	–0.4 (1.9)	[–4.0, 3.4]
Stomach (*N* = 10)	*D*_0.5cc_	0.1 (2.2)	[–4.4, 3.3]	0.3 (3.1)	[–4.5, 5.6]
	*D*_10cc_	–0.2 (1.1)	[–1.4, 2.5]	–0.2 (1.4)	[–1.5, 3.6]
Duodenum (*N* = 11)	*D*_0.5cc_	1.0 (1.7)	[–0.8, 4.9]	1.3 (2.5)	[–1.4, 7.0]
	*D*_5cc_	0.8 (1.9)	[–1.2, 5.7]	1.1 (2.6)	[–2.0, 7.8]
Small bowel (*N* = 8)	*D*_0.5cc_	0.6 (0.9)	[–0.4, 2.3]	0.9 (1.3)	[–0.4, 3.4]
	*D*_5cc_	0.7 (0.7)	[–0.7, 1.5]	1.1 (1.0)	[–0.7, 2.3]
Large bowel (*N* = 11)	*D*_0.5cc_	0.6 (0.9)	[–0.5, 2.2]	1.0 (1.2)	[–0.8, 2.7]
	*D*_20cc_	0.4 (1.0)	[–0.6, 2.4]	0.4 (1.3)	[–1.3, 3.0]
Rectum (*N* = 3)	*D*_1%_	0.2 (0.2)	[0.0, 0.5]	0.3 (0.3)	[0.0, 0.6]
	*D*_10%_	0.0 (0.3)	[–0.3, 0.2]	0.0 (0.4)	[–0.4, 0.3]
	*D*_35%_	–0.3 (0.3)	[–0.6, 0.0]	–0.3 (0.3)	[–0.6, 0.0]
Bladder (*N* = 3)	*D*_1%_	0.1 (0.2)	[–0.2, 0.3]	0.2 (0.1)	[0.1, 0.3]
	*D*_20%_	–0.2 (0.8)	[–1.1, 0.3]	–0.1 (0.8)	[–1.1, 0.4]
	*D*_50%_	–0.5 (1.6)	[–2.4, 0.7]	–0.6 (1.7)	[–2.5, 0.7]
Penile bulb (*N* = 2)	*D*_mean_	0.0 (0.4)	[–0.3, 0.2]	0.1 (0.4)	[–0.2, 0.4]
Femur head L (*N* = 3)	*D*_5%_	–0.5 (1.4)	[–2.1, 0.4]	–0.5 (1.6)	[–2.4, 0.5]
Femur head R (*N* = 3)	*D*_5%_	–0.2 (0.6)	[–0.8, 0.3]	–0.3 (0.9)	[–1.3, 0.3]

*Note*: The mean with one standard deviation (1SD) and range for both physical dose and biologically effective dose (BED) difference are presented. The unit for all the values is percent relative to the dCT‐based dose.

The 1%/1 mm, 2%/2 mm, and 3%/3 mm gamma index for sCT‐based versus dCT‐based doses were 92–100%, 94–100%, and 94–100%, respectively, over the 15 test cases.

## 3 DISCUSSION

This study evaluated the geometric and dosimetric agreement of a sCT‐generation approach as an alternative to a more conventional dCT‐based method that is currently applied on the 0.35 T MRL system. A prototype CNN‐based algorithm was developed for the sCT‐generation from the MRL‐acquired abdominal images. Overall, the geometric and dosimetric agreement between dCT and sCT was good with low MAE and ME of the HU values and average dose differences less than ±1% for all the target volumes and most OARs at different DVH points. In addition to the abdominal region, the presented sCT‐generation approach was applicable in the pelvic but not in the thoracic region.

The HU values of the generated sCT images were pairwise compared to the corresponding dCT. The ME values were close to zero and alternating, which indicates that there was little or no large bias in the sCT HU values. The average MAE values inside the body outline are similar to previously reported values of 30–54 HU for the prostate cases and 62–94 HU for the abdominal cases.^15^ The MAE plotted in 20 HU intervals against the HU values showed that MAE is low around zero (i.e., the HU value of the water) in the HU region that covers majority of the soft tissues, and thus most of human tissues in the abdominal region. However, MAE increases toward the lower and higher HU values, especially for the bony tissues, as the number of voxels in the MAE bins reduces toward the extreme HU regions. Simultaneously, both air and bone are signal‐suppressing tissues in MRI, which makes their intensity on MR images ambiguous.

Considering the geometric agreement, values between 49% and 98% have been reported for DSC_bone_ of the reference CT and sCT for non‐CNN‐based approaches in the pelvis and the brain.^13^ Most of our DSC scores were at the lower end of the reported interval, which is partly explained by smaller bony structures such as the ribs not appearing in the sCT volumes, as we visually observed (see Figure 1). However, the lower DSC scores could also be expected due to our study design; the co‐registration between the testing pCT and the MR images in the ViewRay TPS is not always perfect and differs from the co‐registration in *elastix* prior to the network training. For example, Florkow et al.^20^ have reported a DSC_bone_ of 76% and a DSC_lung_ of 92% for pediatric abdomen with a CNN‐based sCT‐generation in contrast to our DSC_bone_ of 47% and DSC_lung_ of 82%, for the abdominal cases. Yet, Florkow et al.^20^ used a single method to deformably co‐register the MR images with training pCT and testing pCT (prior the sCT‐generation) to enhance dose differences originating solely from the HU differences.

The DSC score also depends on the volume of the masked area favoring larger volumes, so that the DSC_bone_ can be higher for bony areas based solely on the volume size. The different contrast in CT and MRI can also result in differing edge detection in CNN, and thus, in differing tissue volumes. For example, we could visually observe a voxel‐scale difference at the borders of the thresholded bone masks. In addition, image registrations tend to focus on the broader picture and small volumes may be imperfectly registered. Together with registration imperfections and other issues such as partial volume effects, small (signal‐suppressing) volumes on the MRI are difficult for a CNN algorithm to learn properly. The geometric agreement between the dCT and sCT could most likely be improved with more training data and better presentation of the ribs, but only if also the used dCT are in good correspondence with the input MR images.

In the current clinical practice on the MRL, the daily air pockets are delineated on both MRI and dCT. Because the air pockets typically differ between the pCT and the daily anatomy, some differences could also be present between the daily MRI and the corresponding dCT. Thus, the dCT‐contoured pockets are set to water and the MRI‐contoured pockets are set to air—with a priority over the dCT pockets—in the RED map for the daily dose calculation. In this study, we performed RED overwrite with water for air voxels in dCT within the body outline, if no corresponding air was in sCT, before the geometric evaluation. We chose a generic water overwrite as the water RED is very close to the RED of the soft tissues and the bowel content and was in line with the clinical practice. The RED overwrite affects the geometric agreement metrics as clearly visible for the MAE in the 20 HU intervals. However, it also compensates for differences not caused by the performance of the sCT‐generation algorithm and allows the comparison of the dCT and sCT approaches.

The ViewRay 0.35 T MRL system offers one vendor‐dictated MR sequence for the RT dose calculation, whose parameters the user can change to a limited extent. For example, the slice thickness and the number of slices only have a few options. In our data, the MR image quality is sometimes compromised, for example, some high‐intensity artifacts were present, and the ribs were hardly visible to the human eye. Some MRI‐only solutions use dedicated sCT‐MRI sequences such as DIXON and ultra‐short echo time sequences, which could distinguish bones better from other tissues except for nearby air.^2^ From the aspect of IGRT, the exact bone presentation is less critical in MRI‐guided workflow as compared to a standard CT‐based workflow, because the RT alignment is not performed based on bony anatomy. Furthermore, the photon irradiation is more forgiving for RED heterogeneities than for instance protons. As the ribs represent only small structures, their impact on the dose distribution is probably minor. Still, a better representation of ribs is a subject to consider in the future development of the abdominal algorithm.

The accumulated dCT and sCT doses are overall in good agreement with the average relative dose differences within 1% for all the target volumes and most OARs at the evaluated DVH points. However, there are some exceptions with larger dose differences over all the DVH points, the maximum difference being around 4% for the target volumes and 8% for the OARs in BED. The maximum absolute dose differences for PTVs are 1.6 and 6.1 Gy in physical dose and BED, respectively, and for OARs 0.9 and 3.5 Gy in physical dose and BED, respectively. The biggest differences are observed in the near‐minimum doses of the target volumes, for example, *D*_99%_ for the abdominal PTVs, and the near‐maximum doses of OARs *D*_0.03cc_ for the spinal cord, and *D*_0.05cc_ for the duodenum and the stomach. The biggest differences can be expected in these DHV points, because the near‐minimum doses of the target volumes are positioned at steep DVH gradients, and thus, small changes in the DVH shape can result in large absolute deviations. In turn, the small near‐maximum volumes (<0.1 cc) are more sensitive to dosimetric changes than larger volumes.

The dose differences were calculated relative to the dCT‐based dose, which are close to the prescribed doses for the targets (i.e., PTV, CTV, and GTV). For the OARs, the dCT‐based doses are much smaller, and hence larger relative deviations between the sCT and dCT calculations are expected, especially for the near‐maximum doses. In addition, the relative MC calculation uncertainty increases as the dose decreases.^39^ However, large relative deviations can have a relatively smaller clinical relevance for the OARs than the target volumes. For example, the large BED difference at *D*_0.5cc_ of the stomach (–4.5%) and the esophagus (–4.0%) both corresponded to an absolute difference of merely –0.01 Gy. The sCT‐based doses were on average lower than the dCT‐based doses at the evaluated DHV points. Furthermore, the BED differences of the accumulated doses show that the RT fractions do not compensate for under‐ or over‐dosage but rather enhances the already existing deviations. The higher BED differences could be explained by the oART workflow, where the previously delivered doses are not currently considered during daily optimization and adaptation of the RT plan. The dose accumulation diminished the most extreme dose differences in comparison to individual daily doses, but the mean differences remained fairly same.

The largest deviation in both target and OAR doses occurred for three patients with different prescribed doses: (1) 50 Gy in five fractions to adrenal gland; (2) 15 Gy in three fractions to liver; and (3) 15 Gy in five fractions to liver. We did not find a consequent correlation in the cases with the largest deviation, but they could be due to a target volume adjacent to the ribs or a lung interface, a target volume surrounded by air pockets, very low DSC_bone_ around 40%, or a bias in ME larger than ±10 HU. The dose deviations for the prostate cases are overall very moderate (less than ±0.5%). Overall, the DVH deviation values are similar to earlier reported 0%–3% for CNN‐based approaches,^15^ whereas differences less than 2% for PTVs have been considered acceptable for MRI‐only based dose calculations.^40^ The pass rates of the gamma indices for the sCT‐based versus dCT‐based dose comparison with 1%/1 mm, 2%/2 mm, and 3%/3 mm criteria are equal or higher than 92% and similar to previously reported.^15^ In these terms, the dose differences from our study seem acceptable.

There were more men in both the training and the clinical testing groups, however, we did not observe worse results for women as compared to men. The upper abdominal region is very similar for men and women; thus, we believe the algorithm works equally well for both genders. In addition, the patient material had a high average age, but this should correspond with the expected target group. Our study included data that covered several anatomic regions, yet a limited number of patients for each different anatomy. Especially, there were very few lung and pelvic cases in both training and testing groups. The low number of cases make the performance of the sCT algorithm more uncertain for these anatomies and is reflected in the corresponding geometric and dosimetric agreement. To improve the prototype algorithm in these anatomical regions would require access to more training data to ensure that all anatomies are well represented. With sufficient data, the presented sCT‐generation algorithm could be expanded to other sequences and anatomical regions. For example, in head‐and‐neck region, a sCT‐based approach has previously produced accurate dose distributions for offline MRI‐only RTP.^41^

One of our aims for using an sCT‐based approach was to eliminate the image registration between the pCT scan and the daily MRIs. This could remove the uncertainty that arises during the co‐registration, as well as the potential problems that require additional manual corrections, such as subsequent ED overwrites. However, some image registration between pCT and MR images is needed prior to the training of the CNN‐based sCT‐generation network. Our study used deformable registrations also for the dose accumulation, which is accompanied with uncertainties, too. We deemed the dose deformation uncertainties acceptable, as this study only compared the sCT‐ and dCT‐based methods relative to each other and the same deformations are applied to both the dCT and sCT dose distributions.

In this study, the sCT‐based dose distributions were calculated in the ViewRay TPS similarly to the dCT‐based dose distribution. However, this required manual export and import of the images, and manual anonymization and de‐anonymization of the data. To incorporate the sCT‐generation approach into clinical workflow would require adding an automatic export/import patch to the ViewRay system. The sCT‐generation could be achieved with a locally installed server or cloud‐based processing, likewise to offline MRI‐only workflows currently available.

Our study did not cover all aspects of implementing an sCT‐based workflow into clinical practice, for example, the time efficiency of the approach. In general, a sCT volume takes approximately from 2 to 4 min to generate on a GPU depending on the number of slices in MRI, similar to other abdominal networks.^20^, ^25^ The current clinically applied deformable registration for obtaining the dCT takes a few seconds, if the registration is straight forward and does not require corrections. However, the sCT‐generation time could probably be reduced considerably in a subsequent version optimized for oART on the MRL or the sCT‐generation could be performed on the background while re‐contouring or both. The suggested MRI‐only approach could remove the manual corrections; hence, it could reduce overall treatment times and patient discomfort. This has the potential to increase the patient throughput and correspondence of the patient anatomy between the daily MRI and while beam‐on. Simultaneously, user‐dependent variation between patients and fractions due to the manual corrections could be reduced, and the reproducibility of the treatment increased.

## 5 CONCLUSION

Overall, dCT and sCT were in good agreement with average dose differences at different DVH points less than ±1% for all the target volumes and most OARs. Thus, the presented MRI‐only based method is a promising alternative to the current clinical practice on the 0.35 T MRL systems in the abdominal region.

## AUTHOR CONTRIBUTIONS

*Acquisition, analysis, and interpretation of data; drafting the manuscript*: Katri Nousiainen. *Acquisition of data; critical revision of manuscript*: Grichar Valdes Santurio. *Acquisition of data (algorithm training)*: Nils Lundahl. *Critical revision of manuscript*: Rickard Cronholm and Carl Siversson. *Study design; interpretation of data; critical revision of manuscript*: Jens M. Edmund.

## CONFLICTS OF INTEREST

Nils Lundahl, Rickard Cronholm, and Carl Siversson are employed at Spectronic Medical AB. The authors have no other conflicts of interest to declare.

## ETHICS STATEMENT

The ethical approval for this study was granted by the Danish National Committee on Health Research Ethics (approval D1829717 and supplement 85157). The study was performed in line with the principles of the Declaration of Helsinki.

## Supporting information

Supp Information

## References

[1] Das IJ , McGee KP , Tyagi N , Wang H . Role and future of MRI in radiation oncology. Br J Radiol. 2019;92:20180505. 30383454 10.1259/bjr.20180505 PMC6404845

[2] Chandarana H , Wang H , Tijssen RHN , Das IJ . Emerging role of MRI in radiation therapy. J Magn Reson Imaging. 2018;48(6):1468‐1478. 30194794 10.1002/jmri.26271 PMC6986460

[3] Hall WA , Paulson E , Li XA , et al. Magnetic resonance linear accelerator technology and adaptive radiation therapy: an overview for clinicians. CA Cancer J Clin. 2022;72(1):34‐56. 34792808 10.3322/caac.21707 PMC8985054

[4] Hall WA , Paulson ES , van der Heide UA , et al. The transformation of radiation oncology using real‐time magnetic resonance guidance: a review. Eur J Cancer. 2019;122:42‐52. 31614288 10.1016/j.ejca.2019.07.021 PMC8447225

[5] Thorwarth D , Low DA . Technical challenges of real‐time adaptive MR‐guided radiotherapy. Front Oncol. 2021;11:634507. 33763369 10.3389/fonc.2021.634507 PMC7982516

[6] McNair HA , Wiseman T , Joyce E , Peet B , Huddart RA . International survey; current practice in on‐line adaptive radiotherapy (ART) delivered using magnetic resonance image (MRI) guidance. Tech Innov Patient Support Radiat Oncol. 2020;16:1‐9. 32995576 10.1016/j.tipsro.2020.08.002 PMC7501460

[7] Chin S , Eccles CL , McWilliam A , et al. Magnetic resonance‐guided radiation therapy: a review. J Med Imaging Radiat Oncol. 2020;64(1):163‐177. 31646742 10.1111/1754-9485.12968

[8] Owrangi AM , Greer PB , Glide‐Hurst CK . MRI‐only treatment planning: benefits and challenges. Phys Med Biol. 2018;63(5):05TR01. 10.1088/1361-6560/aaaca4 PMC5886006 29393071

[9] Winkel D , Bol GH , Kroon PS , et al. Adaptive radiotherapy: the Elekta unity MR‐linac concept. Clin Transl Radiat Oncol. 2019;18:54‐59. 31341976 10.1016/j.ctro.2019.04.001 PMC6630157

[10] Klüter S . Technical design and concept of a 0.35 T MR‐linac. Clin Transl Radiat Oncol. 2019;18:98‐101. 31341983 10.1016/j.ctro.2019.04.007 PMC6630153

[11] Kraus KM , Jäkel O , Niebuhr, NI , Pfaffenberger A . Generation of synthetic CT data using patient specific daily MR image data and image registration. Phys Med Biol. 2017;62(4):1358‐1377. 28114107 10.1088/1361-6560/aa5200

[12] Mannerberg A , Persson E , Jonsson J , et al. Dosimetric effects of adaptive prostate cancer radiotherapy in an MR‐linac workflow. Radiat Oncol. 2020;15(1):1‐9. 32650811 10.1186/s13014-020-01604-5 PMC7350593

[13] Edmund JM , Nyholm T . A review of substitute CT generation for MRI‐only radiation therapy. Radiat Oncol. 2017;12:28. 28126030 10.1186/s13014-016-0747-y PMC5270229

[14] Johnstone E , Wyatt JJ , Henry AM , et al. Systematic review of synthetic computed tomography generation methodologies for use in magnetic resonance imaging‐only radiation therapy. Int J Radiat Oncol Biol Phys. 2018;100(1):199‐217. 29254773 10.1016/j.ijrobp.2017.08.043

[15] Spadea MF , Maspero M , Zaffino P , Seco J . Deep learning based synthetic‐CT generation in radiotherapy and PET: a review. Med Phys. 2021;48(11):6537‐6566. 34407209 10.1002/mp.15150

[16] Chen S , Qin A , Zhou D , Yan D . U‐net‐generated synthetic CT images for magnetic resonance imaging‐only prostate intensity‐modulated radiation therapy treatment planning. Med Phys. 2018;45(12):5659‐5665. 30341917 10.1002/mp.13247

[17] Dinkla AM , Florkow MC , Maspero M , et al. Dosimetric evaluation of synthetic CT for head and neck radiotherapy generated by a patch‐based three‐dimensional convolutional neural network. Med Phys. 2019;46(9):4095‐4104. 31206701 10.1002/mp.13663

[18] Liu Y , Lei Y , Wang Y , et al. Evaluation of a deep learning‐based pelvic synthetic CT generation technique for MRI‐based prostate proton treatment planning. Phys Med Biol. 2019;64(20):205022. 31487698 10.1088/1361-6560/ab41af PMC7765705

[19] Kazemifar S , Barragán Montero AM , Souris K , et al. Dosimetric evaluation of synthetic CT generated with GANs for MRI‐only proton therapy treatment planning of brain tumors. J Appl Clin Med Phys. 2020;21(5):76‐86. 32216098 10.1002/acm2.12856 PMC7286008

[20] Florkow MC , Guerreiro F , Zijlstra F , et al. Deep learning‐enabled MRI‐only photon and proton therapy treatment planning for paediatric abdominal tumours. Radiother Oncol. 2020;153:220‐227. 33035623 10.1016/j.radonc.2020.09.056

[21] Groot Koerkamp ML , de Hond YJM , Maspero M , et al. Synthetic CT for single‐fraction neoadjuvant partial breast irradiation on an MRI‐linac. Phys Med Biol. 2021;66(8):085010. 10.1088/1361-6560/abf1ba 33761491

[22] Farjam R , Nagar H , Kathy Zhou X , Ouellette D , Chiara Formenti S , DeWyngaert JK . Deep learning‐based synthetic CT generation for MR‐only radiotherapy of prostate cancer patients with 0.35 T MRI linear accelerator. J Appl Clin Med Phys. 2021;22(8):93‐104. 10.1002/acm2.13327 PMC8364266 34184390

[23] Fu J , Singhrao K , Cao M , et al. Generation of abdominal synthetic CTs from 0.35T MR images using generative adversarial networks for MR‐only liver radiotherapy. Biomed Phys Eng Express. 2020;6(1):015033. 33438621 10.1088/2057-1976/ab6e1f

[24] Li X , Yadav P , McMillan AB . Synthetic computed tomography generation from 0.35T magnetic resonance images for magnetic resonance‐only radiation therapy planning using perceptual loss models. Pract Radiat Oncol. 2022;12(1):e40‐e48. 34450337 10.1016/j.prro.2021.08.007 PMC8741640

[25] Cusumano D , Lenkowicz J , Votta C , et al. A deep learning approach to generate synthetic CT in low field MR‐guided adaptive radiotherapy for abdominal and pelvic cases. Radiother Oncol. 2020;153:205‐212. 33075394 10.1016/j.radonc.2020.10.018

[26] Klein S , Staring M , Murphy K , Viergever MA , Pluim JP . Elastix: a toolbox for intensity‐based medical image registration. IEEE Trans Med Imaging. 2010;29(1):196‐205. 19923044 10.1109/TMI.2009.2035616

[27] Shamonin DP , Bron EE , Lelieveldt BP , Smits M , Klein S , Staring M . Fast parallel image registration on CPU and GPU for diagnostic classification of Alzheimer's disease. Front Neuroinform. 2014;7:50. 24474917 10.3389/fninf.2013.00050 PMC3893567

[28] Li W , Wang G , Fidon L , Ourselin S , Cardoso MJ , Vercauteren T . On the compactness, efficiency, and representation of 3D convolutional networks: brain parcellation as a pretext task. In: Niethammer M , Styner M , Aylward S , et al. Information Processing in Medical Imaging. IPMI 2017. Lecture Notes in Computer Science. Vol. 10265. Cham: Springer; 2017:348‐360.

[29] Cronholm RO , Karlsson A , Siversson C . MRI Only Radiotherapy Planning Using the Transfer Function Estimation Algorithm. 2020. Accessed July 5, 2022. http://www.spectronic.se/files/Whitepaper_TFE_202106.pdf

[30] Edmund JM , Andreasen D , Van Leemput K . Cone beam computed tomography based image guidance and quality assessment of prostate cancer for magnetic resonance imaging‐only radiotherapy in the pelvis. Phys Imaging Radiat Oncol. 2021;18:55‐60. 34258409 10.1016/j.phro.2021.05.001 PMC8254192

[31] Andreasen D , Van Leemput K , Hansen RH , Andersen JA , Edmund JM . Patch‐based generation of a pseudo CT from conventional MRI sequences for MRI‐only radiotherapy of the brain Med Phys. 2015;42(4):1596‐1605. 25832050 10.1118/1.4914158

[32] International Commission on Radiation Units and Measurement (ICRU) . Photon, Electron, Proton and Neutron Interaction Data for Body Tissues. ICRU Report 46. ICRU; 1992.

[33] Fedorov A , Beichel R , Kalpathy‐Cramer J , Finet J , Fillion‐Robin J . 3D Slicer as an image computing platform for the quantitative imaging network. Magn Reson Imaging. 2012;30(9):1323‐1341. 22770690 10.1016/j.mri.2012.05.001 PMC3466397

[34] Florkow MC , Zijlstra F , Willemsen K , et al. Deep learning‐based MR‐to‐CT synthesis: the influence of varying gradient echo‐based MR images as input channels. Magn Reson Med. 2020;83(4):1429‐1441. 31593328 10.1002/mrm.28008 PMC6972695

[35] International Commission on Radiation Units and Measurement (ICRU) . Prescribing, Recording, and Reporting Photon‐Beam Intensity Modulated Radiation Therapy (IMRT). ICRU Report 83. ICRU; 2010.

[36] Joiner M , van der Kogel A . Basic Clinical Radiobiology. 5th ed. Boca Raton, FL: CRC Press; 2019. Table 14.2.

[37] Low DA , Harms WB , Mutic S , Purdy JA . A technique for the quantitative evaluation of dose distributions. Med Phys. 1998;25(5):656‐661. 9608475 10.1118/1.598248

[38] Pinter C , Lasso A , Wang A , Jaffray D , Fichtinger G . SlicerRT: radiation therapy research toolkit for 3D Slicer. Med Phys. 2012;39(10):6332‐6338. 23039669 10.1118/1.4754659

[39] Chetty IJ , Rosu M , Kessler ML , et al. Reporting and analyzing statistical uncertainties in Monte Carlo‐based treatment planning. Int J Radiat Oncol Biol Phys. 2006;65(4):1249‐1259. 16798417 10.1016/j.ijrobp.2006.03.039

[40] Korsholm ME , Waring LW , Edmund JM . A criterion for the reliable use of MRI‐only radiotherapy. Radiat Oncol. 2014;9(1):1‐7. 24405515 10.1186/1748-717X-9-16 PMC3909342

[41] Palmér E , Karlsson A , Nordström F , et al. Synthetic computed tomography data allows for accurate absorbed dose calculations in a magnetic resonance imaging only workflow for head and neck radiotherapy. Phys Imaging Radiat Oncol 2021;17:36‐42. 33898776 10.1016/j.phro.2020.12.007 PMC8058030

