# Supplementary material for: Evaluation of MRI‐only based online adaptive radiotherapy of abdominal region on MR‐linac
Source: J Appl Clin Med Phys. 2022 Nov 8;24(3):e13838. doi: 10.1002/acm2.13838 (PMC10018672; doi:10.1002/acm2.13838)
Supplement: Supplementary file 1 — Supp Information [file ACM2-24-e13838-s001.pdf]

Supplementary Material

Table S1 Dose differences between daily dCT and sCT for target volumes at dose-volume-histogram (DVH) points. The mean with one standard deviation (1SD) and range for both physical dose and biologically effective dose (BED) difference are presented. The values are given in % relative to the dCT-based dose.

			**Physical dose difference [%]**		**BED difference [%]**	
**Cases**	**Target**	DVH point	mean (1SD)	[min, max]	mean (1SD)	[min, max]
**All**	**PTV (N=108)**	$D_{2\%}$	0.4 (0.9)	[−1.5, 4.9]	0.5 (1.4)	[−2.2, 7.6]
		$D_{50\%}$	0.4 (0.8)	[−2.2, 4.2]	0.5 (1.2)	[−3.2, 6.4]
		$D_{98\%}$	0.3 (0.9)	[−2.8, 4.5]	0.4 (1.3)	[−3.7, 6.0]
**SBRT**	**PTV (N=68)**	$D_{99\%}$	0.5 (1.2)	[−2.5, 4.3]	0.7 (1.6)	[−3.3, 5.7]
	**GTV (N=68)**	$D_{0.1cc}$	0.4 (1.2)	[−2.5, 4.9]	0.6 (1.9)	[−3.7, 7.6]
		D_{mean}	0.5 (1.1)	[−2.1, 4.5]	0.8 (1.6)	[−2.9, 6.8]
		$D_{99\%}$	0.4 (1.2)	[−3.1, 3.7]	0.5 (1.7)	[−4.4, 5.7]
**Prostate**	**PTV (N=40)**	$D_{2\%}$	0.1 (0.4)	[−0.6, 1.3]	0.1 (0.6)	[−0.8, 1.6]
		$D_{50\%}$	0.2 (0.3)	[−0.3, 1.3]	0.2 (0.4)	[−0.4, 1.6]
		$D_{98\%}$	0.2 (0.4)	[−0.4, 1.3]	0.2 (0.5)	[−0.5, 1.6]
	**CTV (N=40)**	$D_{98\%}$	0.2 (0.3)	[−0.4, 1.1]	0.2 (0.4)	[−0.5, 1.4]

PTV = planning target volume, GTV = gross tumor volume, CTV = clinical target volume, cc = cubic centimeter, SBRT = stereotactic body radiation therapy

Table S2 Dose differences between daily dCT and sCT for organs-at-risk at dose-volume-histogram (DVH) points. The mean with one standard deviation (1SD) and range for both physical dose and biologically effective dose (BED) difference are presented. The values are given in % relative to the dCT-based dose.

		**Physical dose difference [%]**		**BED difference [%]**	
**Structure**	DVH point	mean (1SD)	[min, max]	mean (1SD)	[min, max]
**Spinal cord (N=46)**	$D_{0.03cc}$	0.9 (2.4)	[−3.6, 8.7]	1.2 (3.3)	[−5.0, 12.0]
**Kidney L (N=40)**	$D_{10\%}$	0.2 (1.2)	[−3.7, 3.4]	0.3 (1.4)	[−4.0, 3.5]
	$D_{25\%}$	0.3 (1.5)	[−4.8, 3.4]	0.4 (1.7)	[−4.9, 4.0]
	D_{mean}	0.2 (1.0)	[−3.5, 2.1]	0.3 (1.2)	[−3.4, 2.8]
**Kidney R (N=37)**	$D_{10\%}$	0.4 (1.6)	[−4.0, 4.6]	0.4 (2.0)	[−4.6, 5.6]
	$D_{25\%}$	0.0 (1.7)	[−3.9, 3.9]	0.0 (2.1)	[−5.1, 4.4]
	D_{mean}	0.1 (1.5)	[−2.3, 4.5]	0.3 (1.8)	[−2.9, 5.9]
**Pancreas (N=13)**	D_{mean}	1.0 (1.5)	[−0.4, 4.8]	1.6 (2.6)	[−1.1, 8.0]
**Liver (N=40)**	D_{mean}	0.3 (0.5)	[−0.6, 1.2]	0.6 (0.9)	[−0.7, 2.5]
**Heart (N=11)**	$D_{0.03cc}$	0.0 (1.9)	[−3.7, 2.2]	0.1 (3.0)	[−5.7, 3.5]
	D_{15cc}	−0.7 (1.7)	[−4.5, 1.2]	−0.9 (2.2)	[−5.7, 1.7]
**Great vessels (N=65)**	$D_{0.5cc}$	0.4 (1.5)	[−1.9, 6.8]	0.6 (2.2)	[−2.3, 10.3]
**Esophagus (N=38)**	$D_{0.5cc}$	−0.1 (2.1)	[−6.6, 4.2]	0.0 (2.6)	[−6.9, 6.5]
**Stomach (N=41)**	$D_{0.5cc}$	0.3 (2.1)	[−6.5, 3.9]	0.5 (2.8)	[−6.7, 6.2]
	D_{10cc}	−0.1 (1.7)	[−5.5, 3.7]	−0.2 (2.2)	[−5.6, 4.6]
**Duodenum (N=48)**	$D_{0.5cc}$	0.9 (2.1)	[−1.8, 8.4]	1.4 (3.0)	[−2.8, 11.9]
	D_{5cc}	0.8 (2.1)	[−2.7, 6.7]	1.1 (2.8)	[−3.0, 9.3]
**Small bowel (N=34)**	$D_{0.5cc}$	0.8 (1.2)	[−2.3, 3.0]	1.2 (1.6)	[−2.4, 4.6]
	D_{5cc}	0.5 (1.0)	[−1.5, 3.9]	0.8 (1.3)	[−1.6, 5.3]
**Large bowel (N=48)**	D_{5cc}	0.7 (1.6)	[−2.6, 6.6]	0.9 (2.2)	[−4.1, 7.7]
	D_{20cc}	0.3 (1.3)	[−2.5, 3.4]	0.2 (1.7)	[−3.9, 4.1]
**Rectum (N=45)**	$D_{1\%}$	0.1 (0.7)	[−1.8, 1.7]	0.1 (0.8)	[−2.2, 2.1]
	$D_{10\%}$	0.0 (0.5)	[−0.8, 1.6]	0.0 (0.6)	[−1.0, 2.1]
	$D_{35\%}$	−0.2 (0.5)	[−1.9, 1.1]	−0.3 (0.6)	[−2.1, 1.4]
**Bladder (N=45)**	$D_{1\%}$	0.2 (0.4)	[−0.7, 1.2]	0.2 (0.5)	[−0.9, 1.6]
	$D_{50\%}$	0.1 (0.6)	[−2.4, 1.3]	0.1 (0.7)	[−2.8, 1.6]
	D_{mean}	0.2 (1.0)	[−2.7, 1.6]	0.2 (1.1)	[−3.0, 1.8]
**Penile bulb (N=40)**	D_{mean}	−0.1 (0.6)	[−1.3, 1.0]	0.0 (0.8)	[−1.5, 1.4]
**Femur head L (N=45)**	$D_{5\%}$	0.1 (0.9)	[−4.1, 1.1]	0.1 (1.1)	[−4.7, 1.4]
**Femur head R (N=45)**	$D_{5\%}$	0.0 (0.8)	[−3.1, 1.1]	0.0 (1.1)	[−4.4, 1.5]

cc = cubic centimeter
